# Tick-borne pathogens in questing adults *Dermacentor reticulatus* from the Eastern European population (north-eastern Poland)

**DOI:** 10.1038/s41598-024-51299-x

**Published:** 2024-01-06

**Authors:** Katarzyna Kubiak, Hanna Szymańska, Janina Dziekońska-Rynko, Agnieszka Tylkowska, Małgorzata Dmitryjuk, Ewa Dzika

**Affiliations:** 1 Department of Medical Biology, School of Public Health, Collegium Medicum, University of Warmia and Mazury in Olsztyn, 10-561 Olsztyn, Poland; 2 Department of Zoology, Faculty of Biology and Biotechnology, University of Warmia and Mazury in Olsztyn, 10-957 Olsztyn, Poland; 3 Department of Biology of Animal Environment, Institute of Animal Science, Warsaw University of Life Sciences, 02-786 Warsaw, Poland; 4 Department of Biochemistry, Faculty of Biology and Biotechnology, University of Warmia and Mazury in Olsztyn, 10-719 Olsztyn, Poland

**Keywords:** Parasitology, Pathogens, Risk factors

## Abstract

*Dermacentor reticulatus* is tick species with an expanding geographical range in Europe, which creates the possibility of spreading microorganisms of significant veterinary and medical importance. The study aimed to investigate the prevalence and genetic diversity of *Rickettsia* spp., *Babesia* spp., *Borrelia* spp. and *Anaplasma phagocytophilum* in adult *D. reticulatus* ticks from the Eastern European population in the urban and the natural biotopes of north-eastern Poland. Microorganisms were detected by PCR and identified by DNA sequencing. The overall infection rate of at least one of the pathogens was 29.6%. The predominantly was *Rickettsia* spp. (27.1%) (with *R. raoultii*—9.1%) followed by *Babesia* spp. (2.4%) with *B. canis* (1.5%) as the most frequent. Based on 18S rRNA gene sequence, three *B. canis* genotypes were revealed. The prevalence of *R. raoultii* and *B. canis* was significantly higher in ticks from natural biotopes. The infection rates of *B. afzelii* and *A. phagocytophilum* were determined at 0.9% and 0.3%, respectively. Co-infections were detected in 3.8% of infected ticks. In diagnosing tick-borne diseases in humans, tick-borne lymphadenopathy should not be excluded. The prevalence of different genotypes of *B. canis* suggests differences in the clinical picture of canine babesiosis in the area.

## Introduction

*Dermacentor reticulatus* is the second most abundant tick species after *Ixodes ricinus* in central Europe^1,2^. Until the 1980s, the geographical range of *D. reticulatus* was clearly divided into two main zones: the Western European and the Eastern European populations. In between, there was a large area from the Baltic Sea coast through central Germany, western Poland to the southern border of Hungary where the meadow tick had never been observed^3,4^. Currently, those two populations of *D. reticulatus* (Western and Eastern) are among the most dynamic tick populations in Central Europe^5^. In several European countries (Slovakia, the Czech Republic, the United Kingdom, the Netherlands, and Germany), habitat expansion of *D. reticulatus* was noted^6^, and the northern distribution border in the Baltic countries (Lithuania, Latvia) moved further to the north^7^. On the Polish territory in “the gap” zone, new foci occurrences of this tick species have also been reported^5,8–10^ which leads to closer borders between the western and the eastern *D. reticulatus* populations.

With the expansion of *D. reticulatus* into new areas, the veterinary-medical importance of this species has increased due to the variety of transmitted pathogenic microorganisms^11^. *D. reticulatus* is considered the main vector of the protozoa *Babesia canis* (the etiological agent of canine babesiosis), as well bacteria of the genera *Rickettsia*, *Anaplasma*, *Bartonella*, *Francisella*, *Coxiella burnetii*^2,12^ and tick-borne encephalitis virus^13^. The significance of *D. reticulatus* in the transmission of *Borrelia burgdorferi* s.l. is still unclear^2,12^. Not without significance is also the contribution of *D. reticulatus* ticks to the maintenance of tick-borne pathogens in the environment through the transfer of pathogenic microorganisms between various vertebrate hosts, which are susceptible to infection and serve as efficient reservoirs^12^.

The eastern population of *D. reticulatus* ticks in Poland is considered a stable population and is the source of newly emerging foci of this species in the zone previously considered free (central and western Poland)^5,9^, and thus the spread of tick-borne microorganisms of significant veterinary and medical importance. The aim of the study was to assess the prevalence and diversity of tick-borne protozoa (*Babesia* spp.) and bacteria (*Rickettsia* spp., *A. phagocytophilum*, *Borrelia* spp.) in adult *D. reticulatus* ticks from the Eastern European population in the urban and the natural biotopes of north-eastern Poland.

## Material and methods

### Study area and tick collection

**Figure 1:**
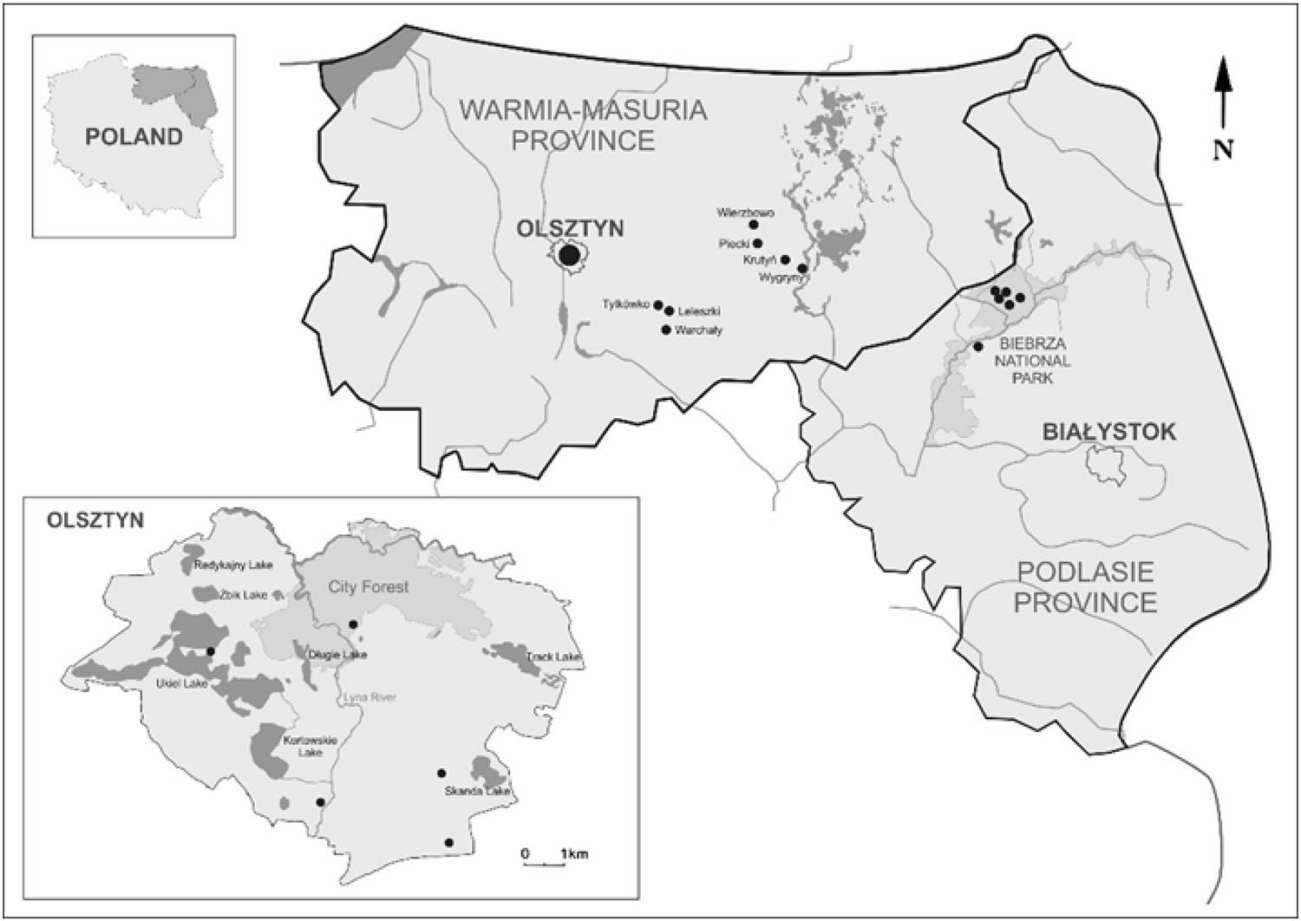
*Dermacentor reticulatus* tick collection sites located in north-eastern Poland. The map was designed in CorelDRAWX5 based on Google Maps (https://www.google.pl/maps).

Questing adult *D. reticulatus* ticks (n = 886, 587 females, 299 males) were collected between March-June in 2016 and 2017. The collection sites were located in urbanized areas (within the administrative borders of the city of Olsztyn) (n = 395) and in natural biotopes of the central part of the Warmia and Mazury region (n = 87) as well as in the Biebrza National Park (n = 404) (Podlasie region) (Fig. 1) (Supplementary Table S1). Tick collections were performed twice per month in each year of the study during the daytime between 9 a.m. and 4 p.m. using the standard flagging method^14^. In the laboratory, specimens were morphological identified by species and sex using taxonomic key^15^ and were preserved individually in 70% ethanol at 4 °C.

### DNA extraction

The genomic DNA from *D. reticulatus* ticks was extracted using the ammonia method^16^. Before DNA extraction, ticks preserved in 70% ethanol were air-dried and then separately cut and crushed using a sterile mortar in 0.7 M ammonium hydroxide (NH_4_OH). The obtained DNA lysates were stored at − 20 °C for further molecular analysis.

### Pathogen DNA detection

Tick DNA samples were used to detect tick-borne microorganisms. For the detection of DNA of *Babesia* spp., a nested PCR (nPCR) reaction targeting the 18S rRNA gene using the primers CRYPTO F/CRYPTO R and Bab GF2/Bab GR2^17–19^ was used (Table 1). The presence of *Rickettsia* spp. in tick genomic DNA samples was confirmed by using a set of primers (CS409/Rp1258)^20^ specific to the fragment of the citrate synthase (*gltA*) gene (Table 1). *A. phagocytophilum* DNA was detected using the nPCR with two sets of primers (ge3a/ge10r and ge9f/ge2) targeting fragments of the 16S rRNA gene^21^ (Table 1). Detection of *B. burgdorferi* s.l. DNA was performed via the nPCR-restriction fragment length polymorphism (RFLP) method using primers 132f/905r and 202f/832r specific to the *flaB* gene^22,23^ (Table 1). The identification of species belonging to the *Borrelia burgdorferi* s. l. complex was based on the restriction patterns obtained by digestion of the inner-PCR product (604 bp) by using the restriction enzyme HpyF3I (*DdeI*) (ThermoFisher Scientific, Waltham, MA, USA)^22,23^.

**Table 1:** Primer sets used for PCR amplification.

Pathogen	Primer name	Primer sequence 5′–3′	Gene target	Product size [bp]	Annealing temperature [°C]	Reference
*Babesia* spp.	CRYPTO F	AACCTGGTTGATCCTGCCAGTAGTCAT	18S rRNA	~ 1200	60	Bonnet et al.^17^, Bonnet et al.^18^, Mierzejewska et al.^19^
	CRYPTO R	GAATGATCCTTCCGCAGGTTCACCTAC				
	BabGF2	GYYTTGTAATTGGAATGATGG		550	59	
	Bab GR2	CCAAAGACTTTGATTTCTCTC				
*Rickettsia* spp.	CS409	CCTATGGCTATTATGCTTGC	*gltA*^1^	769	55	Roux et al.^20^
	Rp1258	ATTGCAAAAAGTACAGTGAACA				
*Anaplasma phagocytophilum*	ge3a	CACATGCAAGTCGAACGGAT TATTC)	16S rRNA	932	55	Massung^21^
	ge10r	TTCCGTTAAGAAGGATCTAATCTCC				
	ge9f	AACGGATTATTCTTTATAG		546	55	
	ge2	CTTGCTGGCAGTATTAAAAGCAGCTCCAGG				
*Borrelia* spp.	132f	TGGTATGGGAGTTCTGG	*flaB*^2^	774	52	Wodecka et al.^23^, Wodecka^22^
	905r	TCTGTCATTGTAGCATCTTT				
	220f	CAGACAACAGAGGGAAAT		604	54	
	823r	TCAAGTCTATTTTGGAAAGCACC				

^1^*gltA*—citrate synthase gene.

^2^*flaB*—flagellin gene.

All amplifications were performed in a total volume of 25 µL of PCR mixture containing 12.5 µL of 2× PCR Master Mix Plus (0.1 U/µL of Taq polymerase supplied in a PCR buffer, 4 mM of MgCl_2_ and 0.5 mM of each dNTPs) (A&A Biotechnology, Gdynia, Poland), 0.5 µL of each primer (10 µM), 2–4 µL of template DNA (in nPCR—1 µL of template DNA or 1 µL of the outer-PCR product) and an appropriate amount of sterile nuclease-free water. DNA of *B. canis* isolated from the blood of an infected dog, *Rickettsia* spp., *A. phagocytophilum* and *B. afzelii* obtained from an infected *I. ricinus* tick (confirmed by sequencing in earlier studies) were used as a positive control. PCR amplicons were visualized on 1.5% agarose gels stained with Midori Green Stain (Nippon Genetics Europe, Düren, Germany) using GelDocXR (Bio-Rad, Hercules, California, USA).

### Identification of pathogen species

To confirm the species of the detected microorganisms, selected PCR products (81 for *Rickettsia* spp., 21 for *Babesia* spp., three for *A. phagocytophilum*, five for *Borrelia* spp.) were purified using the CleanUp purification kit (A&A Biotechnology, Gdynia, Poland) according to the manufacturer’s protocol and sequenced bi-directionally (Macrogen Europe, Amsterdam, the Netherlands) with forward and reverse primers. The obtained nucleotide sequences were edited in BioEdit v. 7.2 software (https://bioedit.software.informer.com, accessed on February 2022) and compared with data registered in the GenBank database (http://www.ncbi.nih.gov/Genbank/index.html, accessed on May 2023) using the BLAST-NCBI program (http://www.ncbi.nlm.nih.gov/BLAST).

Consensus sequences were deposited in the GenBank database and registered under the accession numbers ON660868-870 for the *gltA* gene of *Rickettsia* spp., OR056353, OR064513-518 for the 18S rDNA of *Babesia* spp., OR096226-227 for the 16S rDNA of *A. phagocytophilium* and OR046059-060 for the *flaB* gene of *Borrelia* spp.

The phylogram was constructed using the Maximum Likelihood method based on the Kimura 2-parameter model. The topology of the phylogenetic tree was evaluated using the bootstrap method with 1000 replicates. The phylogenetic analysis was conducted using MEGA X software (https://www.megasoftware.net).

### Statistical analysis

A chi-square test (with post-hoc Bonferroni test) and 95% confidence intervals (95% CI) were used to compare differences in the prevalence of detected microorganisms in the sex of ticks, regions, habitats and years of study. In all analyses, p-values below 0.05 were considered statistically significant. The analysis was conducted using the software package SPSS version 27.0 for Windows (SPSS Inc., Chicago, USA).

## Results

### Total prevalence of pathogens

In north-eastern Poland, the overall infection rate of *D. reticulatus* ticks for at least one of the examined pathogens was 29.6% (262/886) (Table 2). Females (30.2%) and males (28.4%) were infected in a similar proportion (χ^2^_1_ = 0.283, p = 0.595). Significant differences in pathogen prevalence were noted between the years of the study (χ^2^_1_ = 8.515, p = 0.004) (Table 2). The DNA of microorganisms was confirmed in 33.3% and 24.3% of the tested ticks in 2016 and 2017, respectively. The highest prevalence was recorded in *D. reticulatus* ticks collected in Biebrza National Park (39.4%) compared to the central part of the Warmia-Mazury region (28.7%) and the city of Olsztyn (19.7%) (χ^2^_2_ = 36.909, p < 0.001) (Table 2). In the population of *D. reticulatus* from natural areas, the level of infection was almost twice as high as from urban areas, 37.5% and 19.7% (χ^2^_1_ = 33.032, p < 0.001), respectively (Table 2).

**Table 2:** Total infection rate in *Dermacentor reticulatus* ticks by sex, year, region and biotope in northeastern Poland.

	No. (%) of collected	No. (%) of infected	95% CI	p-value*
Sex				
Females	587 (66.3)	177 (30.2)^a^	26.46–34.04	0.595
Males	299 (33.7)	85 (28.4)^a^	23.38–33.91	
Year				
2016	519 (58.6)	173 (33.3)^a^	29.29–37.57	0.004
2017	367 (41.4)	89 (24.3)^b^	19.95–28.97	
Region				
Olsztyn city	395 (44.6)	78 (19.7)^a^	15.93–24.04	< 0.001
Warmia-Mazury region	87 (9.8)	25 (28.7)^ab^	19.54–39.43	
Biebrza National Park	404 (45.6)	159 (39.4)^b^	34.56–44.31	
Biotope				
Natural	491 (55.4)	184 (37.5)^a^	33.18–41.92	< 0.001
Urban	395 (44.6)	78 (19.7)^b^	15.93–24.02	
Total	886 (100)	262 (29.6)	26.28–32.70	

*χ^2^ test, p < 0.05.

^a,b^Different letters mean significant differences (post-hoc Bonferroni test).

### Diversity and genotyping of pathogens

#### *Rickettsia* spp.

*Rickettsia* spp. DNA was the most frequently detected in questing *D. reticulatus* ticks (Table 3). The overall *Rickettsia* spp. infection rate was 27.1% (240/886). The prevalence of this bacteria did not differ between females (27.8%) and males (25.8%). A significant difference was noted between ticks collected in natural biotopes (35.6%) and in urban areas (16.5%) (χ^2^_1_ = 40.797, p < 0.001). *Rickettsia* spp. DNA was the most frequently confirmed in ticks collected in the Biebrza National Park (38.1%), followed by the central part of the Warmia-Mazury region (24.1%) and the city of Olsztyn (16.5%) (χ^2^_2_ = 47.882, p < 0.001). The percentage of *Rickettsia*-positive ticks was significantly higher in 2016 (32.0%) than in 2017 (20.2%) (χ^2^_1_ = 15.210, p < 0.001).

**Table 3:** Pathogen prevalence in *Dermacentor reticulatus* ticks by sex, year, region and biotope in northeastern Poland.

Species	Sex		Year		Region			Biotope		Total infected
	F	M	2016	2017	Olsztyn city	W-M region	BNP	natural	urban	
	No. (%) of infected p-value*									
*Rickettia* spp. (total)	163 (27.8)	77 (25.8)	166 (32.0)	74 (20.2)	65 (16.5)	21 (24.1)	154 (38.1)	175 (35.6)	65 (16.5)	240 (27.1)
	0.523		< 0.001		< 0.001			< 0.001		
*Rickettsia raoultii*	60 (10.2)	21 (7.0)	50 (9.6)	31 (8.4)	25 (6.3)	19 (21.8)	37 (9.2)	56 (11.4)	25 (6.3)	81 (9.1)
*Babesia* spp. (total)	15 (2.6)	6 (2.0)	8 (1.5)	13 (3.5)	11 (2.8)	8 (9.8)	2 (0.5)	10 (2.0)	11 (2.8)	21 (2.4)
	0.612		0.054		< 0.001			0.467		
*Babesia canis*	7 (1.2)	6 (2.0)	7 (1.3)	6 (1.6)	4 (1.0)	8 (9.2)	1 (0.2)	9 (1.8)	4 (1.0)	13 (1.5)
*Babesia microti*	8 (1.3)	0 (0.0)	1 (0.2)	7 (1.9)	7 (1.8)	0 (0.0)	1 (0.2)	1 (0.2)	7 (1.8)	8 (0.9)
	0.043		0.085		0.018			0.024		
*Anaplasma phagocytophilum*	2 (0.3)	1 (0.3)	0 (0.0)	3 (0.8)	2 (0.5)	0 (0.0)	1 (0.2)	1 (0.2)	2 (0.5)	3 (0.3)
	1		0.071		0.696			0.598		
*Borrelia afzeli*	6 (1.0)	2 (0.7)	5 (1.0)	3 (0.8)	2 (0.5)	0 (0.0)	6 (1.5)	6 (1.2)	2 (0.5)	8 (0.9)
	0.724		1		0.221			0.310		

*F -* female, *M -* male, *W-M* - Warmia-Mazury, *BNP - * Biebrza National Park.

*χ^2^ test, p < 0.05.

**Figure 2:**
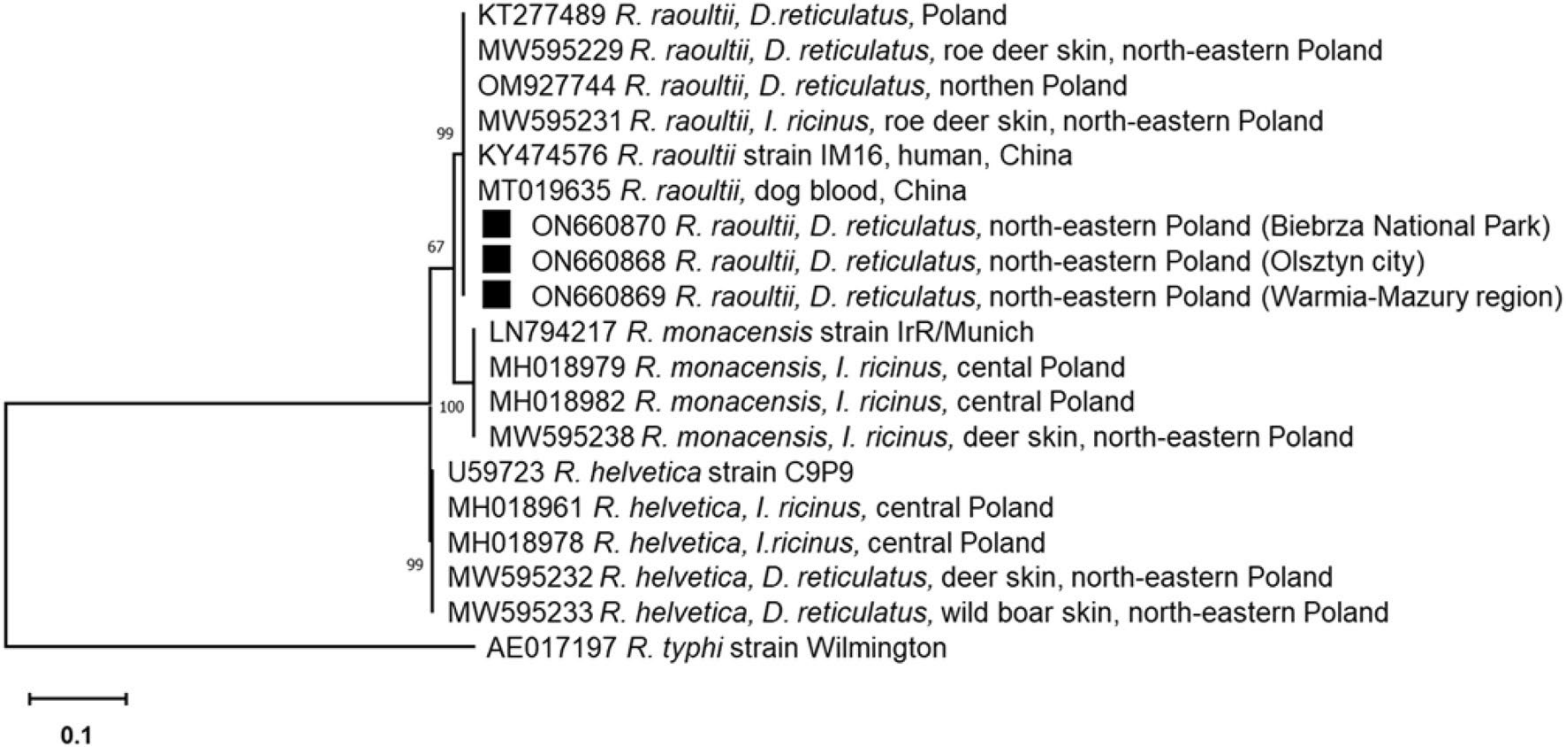
Phylogenetic relationships between *Rickettsia raoultii* identified in the study and accessions from GenBank, based on the sequences of the *gltA* gene of *Rickettsia* spp*.* The phylogram was constructed conducted in MEGA X software (https://www.megasoftware.net) using the Maximum Likelihood method and the Kimura 2-parameter method as a distance method. The percentage of replicate trees in which the associated taxa are clustered together in the bootstrap test (1000 replicates) is shown next to the branches. The tree is drawn to scale, with branch lengths measured in the number of base substitutions per site. The sequences obtained in this study are labelled with black symbols.

Sequence analysis of the partial *gltA* gene indicated the presence of only *R. raoultii* in *D. reticulatus*. All obtained nucleotide sequences (n = 81) were similar and showed 100% identity to *R. raoultii* isolated from the blood of a patient (GenBank: KY474576) and dog (GenBank: MT019635) in China as well as ticks from Poland (Fig. 2).

#### *Babesia* spp.

The total infection rate of *Babesia* spp. in *D. reticulatus* ticks was 2.4% (21/886) (Table 3). The infection rate did not significantly differ between females (2.6%) and males (2.0%) or the years of study (2016–2017) (1.5% and 3.5%, respectively). The highest prevalence of *Babesia* spp. was in the central part of the Warmia-Mazury region (9.8%) (χ^2^_2_ = 23.946, p < 0.001). However, no significant differences between natural biotopes (2.0%) and urban areas (2.8%) (χ^2^_1_ = 0.529, p = 0.467) (Table 3) were noted.

Among infected *Babesia* spp. ticks (n = 21), nucleotide sequences analysis of partial *18S rRNA* gene revealed the presence of *B. canis* and *B. microti*. Statistically significant differences in the occurrence of both species of protozoa were determined in relation to the studied region (χ^2^_2_ = 8.086, p = 0.018), biotope (χ^2^_1_ = 6.390, p = 0.024) and sex of ticks (χ^2^_1_ = 5.169, p = 0.046) (Table 3).

**Figure 3:**
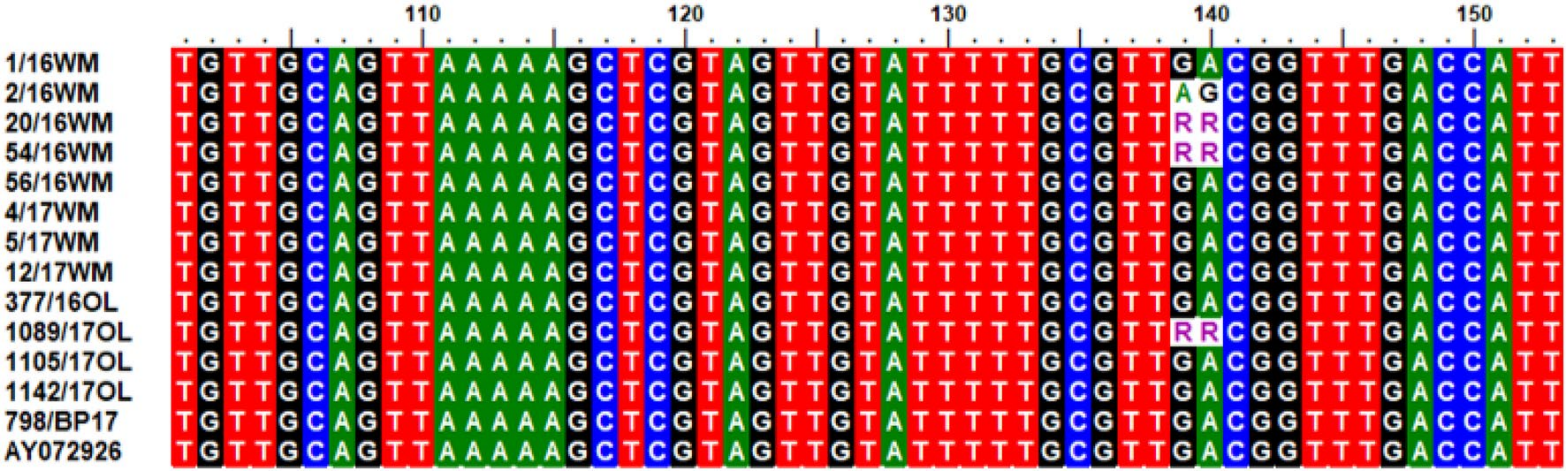
Partial nucleotide sequences of *B. canis* 18S rRNA gene (n = 13) with single nucleotide polymorphisms (SNPs) in positions 139–140 corresponding to positions 609–610 of the reference sequence AY072926 in the GenBank (A—adenine, G—guanine, T—thymine, C—cytosine, RR—double peaks G/A).

*B. canis* was identified in 1.5% of ticks (13/886), including females (1.2%, n = 7) and males (2.0%, n = 6) in all examined years (2016–2017). This species of protozoan was detected mainly in *D. reticulatus* ticks from the central part of the Warmia-Mazury region (9.2%, 8/87). Ticks from both natural and urban biotopes were *B. canis*-positive in similar proportions, 1.8% (n = 9) and 1.0% (n = 4), respectively. All obtained chromatograms of the partial 18S rRNA gene *B. canis* were checked manually. In analyzed sequences at the positions corresponding to the 609–610 nucleotides of the complete *ssrRNA* gene (GenBank: AY072926), two nucleotide substitutions (GA/AG) were observed (Fig. 3). In 69.2% (9/13) of *B. canis* isolates GA dinucleotide (genotype A) was detected. Those sequences were 100% identical to sequences detected in the blood of naturally *B. canis*-infected dogs from Poland (GenBank: EU622793) and from Croatia (GenBank: MK089785), as well as in questing *D. reticulatus* in Poland (GenBank: KT272401). The AG nucleotide combination (genotype B) (Fig. 3) was detected in one *B. canis* positive sample (7.7%, 1/13) and it showed 100% identity to sequences revealed in *B. canis* from the blood of a dog in Poland (GenBank: EU622792) as well *D. reticulatus* from Romania (GenBank: MK836022) and Kazakhstan (GenBank: MK070118). It was found that 23.1% of *B. canis* isolates (3/13) had the GA/AG nucleotide double peak at positions 609–610 of the complete ssrRNA gene (GenBank: AY072926) and belonged to genotype A/B (Fig. 3).

*Babesia microti* were found in 0.9% (8/889) of examined *D. reticulatus* ticks. *B. microti* were detected only in females, mostly collected from an urban area (Olsztyn city) (1.8%, n = 7). Only one tick infected by *B. microti* was from natural areas (Biebrza National Park). All isolates identified as *B. microti* showed 100% identity with the genetic variants included in nonpathogenic *B. microti* Munich type (GenBank: AB071177, AY789075).

#### *Anaplasma phagocytophilium* and *Borrelia* spp.

*Anaplasma phagocythophilum* was identified in 0.3% (3/886) of tick DNA samples at the same proportion in females (0.3%) and males (0.3%) (Table. 3). DNA of *B. burgdorferi* s.l. was detected in 0.9% (8/886) of *D. reticulatus* of females (1%) and males (0.7%) (Table 3). There were no significant differences found in the infection rates of *A. phagocythophilum* or *Borrelia* spp. based on year, region or biotope.

All *A. phagocythophilum*-positive samples were confirmed by nucleotide sequencing. All of them were identical and showed 100% similarity to the sequence of *A. phagocythophilum* derived from the blood of red deer in Czechia (GenBank: EU839849) and of Merino sheep in Germany (GenBank: MZ348273). *Borrelia* spp. in *D. reticulatus* samples were identified as *B. afzelii*. The sequence showed 100% identity to the strains BO23 (GenBank: CP018262) and K78 (GenBank: CP009058) that were detected in symptomatic patients with borreliosis in Germany and Austria.

#### Co-infections of *Rickettsia* spp., *Babesia* spp., *Anaplasma phagocytophilum* and *Borrelia* spp.

Among 267 infected *D. reticulatus* ticks, co-infections of examined microorganisms were recognized in 3.8% (n = 10) of them (Table 4). All noted co-infections were double. Double infections of *R. raoulti*i or *Rickettsia* spp.*/B. canis* and *R*. *rauoltii*/*B. afzelii* were identified in equal proportions. Each of them accounted for 1.5% (n = 4) of the infected ticks (Table 4). Co-infections of *R*. *raoultii*/*B. microti* or *B. afzelii*/*A. phagocytophilum* were detected in only one infected *D. reticulatus* tick (0.4% each). Most co-infections (9/10; 90%) were detected in *D. reticulatus* females (Table 4).

**Table 4:** Co-infection with the pathogens of the genera *Rickettsia*, *Babesia*, *Anaplasma* and *Borrelia* in *Dermacentor reticulatus* ticks from north-eastern Poland.

	No. (%) of infected/sex	95% CI
Mono-infections	252 (96.2)	93.09–98.15
Co-infections	10 (3.8)	1.85–6.91
*Rickettsia raoultii* [*Rickettsia* spp.] + *Babesia canis*	4 [1] (1.5)/F	0.42–3.86
*Rickettsia raoultii* + *Borrelia afzelii*	4 (1.5)/3 F, 1M	0.42–3.86
*Rickettsia raoultii* + *Babesia microti*	1 (0.4)/F	0.01–2.11
*Borrelia afzelii* + *Anaplasma phagocytophilum*	1 (0.4)/F	0.01–2.11
Total infected	262 (100)	

*F* female, *M* male.

## Discussion

The authors’ previous monitoring of tick occurrence in north-eastern Poland demonstrated that *D. reticulatus* is permanently present in both natural biotopes as well in the green areas located in cities^24–27^. The present study focused on molecular evidence of veterinary-medical important pathogens transmitted by questing *D. reticulatus* ticks belonging to the Eastern European population from urban and natural biotopes of a region of north-eastern Poland. The presence of genetic material of all four studied groups of microorganisms (*Rickettsia* spp., *Babesia* spp., *A. phagocytophilum*, *Borrelia* spp.) was found in the study, and almost every third *D. reticulatus* tick (29.6%) was infected with at least one of them, regardless of sex.

From an epidemiological point of view, *D. reticulatus* ticks are less important than *I. ricinus* ticks. In veterinary practice, the most important pathogen transmitted by *D. reticulatus* is *B. canis* protozoa, which causes canine babesiosis^28^. In the current study, *B. canis* was identified in *D. reticulatus* ticks in similar proportions in both urban and natural biotopes. However, the overall level of infection was relatively low (1.4%) compared to ticks from other sites in the eastern macroregion. The prevalence of *B. canis* in ticks from the Eastern European population has been reported to vary from 0 to 6.8%^19,25,29–34^. It is worth underlining that in the current study, most of the *D. reticulatus* infected by *B. canis* were detected in an urban area of the city of Olsztyn in the central part of the Warmia and Mazury region, the oldest area known as endemic for *D. reticulatus*^3,5^. These results confirm the presence and relatively constant level of prevalence *B. canis* (2.5%) in this part of north-eastern Poland, previously established by other authors in the range of 2.3–6%^5,19^.

In spite of a low overall prevalence, the molecular analysis of a fragment of the 18S rRNA gene of *B. canis* indicates the presence of genetically heterogenic genotypes of *B. canis* in examined ticks. In Europe, based on two single nucleotide polymorphisms (SNPs), four *B. canis* genotypes related to GA → AG nucleotide substitutions are present at different rates of prevalence^35–37^. In the present study, among three revealed genotypes, the vast majority of *B. canis* isolates (69.2%) represented genotype A (GA nucleotides). However, the “mixed” A/B genotype (showing the presence of both G and A nucleotides—R/R) has also been recorded. The A/B genotype was the most frequently detected in naturally infected dogs in Poland and other countries from northern Europe^37,38^. The occurrence of genotype A and genotype B (AG nucleotides) of *B. canis* in *D. reticulatus* in north-eastern Poland may suggest differences in the clinical manifestation of canine babesiosis in this area. Adaszek^39^ revealed that the severity of thrombocytopenia in dogs infected with *B. canis* is related to different 18S rRNA genotypes of the pathogen. Genotype B of *B. canis*, also identified in the current study, was found to be more virulent in relation to thrombocytopenia than genotype A.

In 0.9% of examined *D. reticulatus* ticks, the DNA of *B. microti*, considered to be the most common causative agent for human babesiosis, was also detected^40^. Interestingly, it was detected only in females from urban areas. Although the occurrence of DNA of *B. microti* was previously reported in *D. reticulatus*^19,25,30,41^, a role for this tick species as a vector for *B. microti* has not been clearly confirmed. The presence of *B. microti* DNA in questing *D. reticulatus* ticks may be due to feeding on voles (*Microtus* spp.), which have been identified as the main reservoir of *B. microti*^42^.

“Contamination” with the blood of the host is probably also the cause of the detection of *Borrelia* spp. DNA in examined *D. reticulatus* ticks. Although the specific DNA of that bacteria has been detected in *D. reticulatus* in other regions of Poland^19,26,27,30^, their role as a vector was not confirmed. It has been proven that the salivary glands of *Dermacentor* spp. contain proteins (defensins), which are attributed to the role of specific antibiotics in eliminating spirochetes^43^.

The significance of *D. reticulatus* as a vector of *A. phagocytophlium*—the causative agent of human granulocytic anaplasmosis (HGA)^44^ also seems to be insignificant. A meta-analysis of the prevalence and distribution of *A. phagocytophilum* in tick vectors conducted by Karshim^45^ showed that the overall level of infection with this pathogen in questing *D. reticulatus* ticks is very low (0.42%). The current results (0.3%) correspond with the results of other studies on questing *D. reticulatus* ticks belonging to the East European population (0.7–3%)^30,33,46–48^ and confirmed that fact. However, it should be noted that the infection rate of *A. phagocytophilum* in *D. reticulatus* may vary significantly depending on the year of study, the local availability of hosts and the phase of the life cycle (non-feeding/feeding on the host)^26,27,30,33,34,46–51^.

From a medical point of view, *D. reticulatus* is of undeniable importance in the transmission of bacteria representing the genus *Rickettsia*^12^. Although *D. reticulatus* ticks attack humans sporadically^52–54^, it is a main vector of *R. raoultii* and *R. slovaca*. Tick-borne lymphadenopathy (TIBOLA)/Dermacentor-borne necrosis erythema-lymphadenopathy (DEBONEL) or scalp eschar and neck lymphadenopathy after a tick bite (SENLAT) are recently described infection syndromes in humans caused by *R. raoultii* and *R. slovaca* belonging to spotted fever group (SFG) rickettsiae^55,56^. To date, several cases of DEBONEL/TIBOLA have been described in Europe, including in Poland^57–61^. *R. raoultii* is the most commonly detected pathogen in both adult and juvenile *D. reticulatus* ticks in Poland^62^. *R. raoultii* was the only species identified by DNA sequencing in 33% of *Rickettsia*-positive ticks in this study. The frequency of this pathogen in the examined specimens ranged from 6.3 to 21.8% depending on the research region and was much higher in ticks collected from natural biotopes than in urban areas (the city of Olsztyn).The level of occurrence of *R. raoultii* in ticks in north-eastern Poland was comparable to that previously determined by Mierzejewska^19^ in this area (34.2%). A higher infection rate, ranging from 41 to 91.7%, was detected in adult *D. reticulatus* in populations in other parts of north-eastern and eastern Poland^34,63–65^. Mierzejewska^19^, comparing the prevalence of *R. raouliti* between two Polish tick populations (eastern and western) with an expansion zone between them, noted slight differences (42% in the East vs. 52% in the West), but with a clearly increasing gradient from east to west in Poland. A high prevalence of *Rickettsia* spp. in the population of *D. reticulatus* ticks may result from the possibility of transmission not only through the infected host—tick route, but also via the vertical transovarial, transstadial and, probably less frequently, transspermal transmission^56^.

In the case of tick-borne diseases in humans, tick co-infection with several species of pathogenic microorganisms and their co-transmission might have important relevance to public health^66^. In *D. reticulatus* from north-eastern Poland, co-infections were revealed in 3.8% of positive ticks. Considering that only co-infections with *R. raoultii* with *B. canis* were identified (which are not pathogenic for humans) and with *B. afzelii* (which are probably neutralized by defensins produced by *D. reticulatus* ticks), it seems that this tick species does not play an important role as a vector of mixed tick-borne infections in humans that could exacerbate the course of the disease severity and make it difficult to diagnose or treat.

## Conclusion

The current study has confirmed the high prevalence of *R. raoultii* in adult ticks in the eastern population of *D. reticulatus* in north-eastern Poland. The presence of bacteria belonging to the rickettsiae from the spotted fever group indicates that tick-borne lymphadenopathy (DEBONEL/TIBOLA) should not be excluded in the diagnosis of tick-borne diseases. In turn, the relatively high prevalence of *B. canis* with different genotypes suggests differences in the clinical picture of canine babesiosis in this area. The risk of infection with these two pathogens is much higher in the natural biotopes of north-eastern Poland. *D. reticulatus* seems to play a minor role in the transmission of *Borrelia burgdorferi* s.l. and *A. phagocytophilum,* as well as a vector of mixed infections in humans.

### Supplementary Information

Supplementary Table S1.

## Data Availability

The datasets used and analyzed during this study are available from the corresponding author (K.K.) upon reasonable request. The nucleotide sequences have been deposited in the GenBank database under accession numbers: ON660868-870 (*Rickettsia* spp.), OR056353, OR064513-518 (*Babesia* spp.) OR096226-227 (*A. phagocytophilium*) and OR046059-060 (*Borrelia* spp.).
