# Supplementary material for: Tick-borne pathogens in questing adults Dermacentor reticulatus from the Eastern European population (north-eastern Poland)
Source: Sci Rep. 2024 Jan 6;14:698. doi: 10.1038/s41598-024-51299-x (PMC10771447; doi:10.1038/s41598-024-51299-x)
Supplement: Supplementary file 1 — Supplementary Table S1. [file 41598_2024_51299_MOESM1_ESM.docx]

| **Region** | **Locality** | **Geographical coordinates** | **Habitat** |
| --- | --- | --- | --- |
| City of Olsztyn | City Forest | 53°47'51.9"N 20°28'35.0"E | Ecotone (meadow/forest) |
|  | Brzeziny Est. | 53°44'46.9"N 20°27'34.8"E | Open landscape |
|  | Mazurskie Est. | 53°45'39.4"N 20°31'06.1"E | Open landscape |
|  | Tęczowy Las Est. | 53°43'54.2"N 20°29'50.5"E | Ecotone (grassy path/forest) |
|  | Ukiel Lake | 53°46'23.9"N 20°26'25.1"E | Ecotone  (forested areas and lake shore vegetation) |
| Central  Warmia and Mazury | Tylkówko | 53°62'1585"N 20°71'9472"E | Meadow |
|  | Warchały  (Barajnickie Lake) | 53°32'15.6"N 20°49'14.6"E | Ecotone  (forested areas and lake shore vegetation) |
|  | Leleszki | 53^o^37’02.6"N 20°43’17.3"E | Ecotone (grassy path/forest) |
|  | Wierzbowo | 53°48'24.0"N 21°19'25.6"E | Ecotone (grassy path/forest) |
|  | Piecki | 53°48'01.7"N 21°19'33.1"E | Forest landscape |
|  | Krutyń | 53°42'09.0"N 21°26'21.4"E | Forest landscape |
|  | Wygryny | 53°40'15.8"N 21°32'17.1"E | Ecotone (grassy path/forest) |
| Biebrza National Park | Grzędy  car parking | 53°63'17.3"N 22°77'41.5"E | Ecotone (grassy path/forest) |
|  | Grzędy  jogging path | 53°67'75.2"N 22°81'38.2"E | Ecotone (grassy path/forest) |
|  | Grzędy  „Uroczysko -Nowy Świat” | 53°59'53.4"N 22°86'01.7"E | Ecotone (grassy path/forest) |
|  | Duża Łąka | 53°61'46.1"N 22°77'06.6"E | Ecotone (meadow/forest) |
|  | Dział Grabowskiego | 53°59'67.6"N 22°80'41.6"E | Ecotone (meadow/forest) |
|  | Osowiec Twierdza | 53°28'23.0"N 22°39'27.0"E | Ecotone (grassy path/forest) |

Table S1. Characteristics of *Dermacentor reticulatus* tick collection localities in north-eastern Poland
